# Hydroxychloroquine Induces Remission for IgA Nephropathy With Mild to Moderate Proteinuria: A Single-Centered Retrospective Analysis

**DOI:** 10.7759/cureus.53395

**Published:** 2024-02-01

**Authors:** Yixuan Pan, Jingyun Le, Lan Lan, Yaomin Wang, Guangjun Liu, Xiaoqi Shen, Pingping Ren, Jianghua Chen, Fei Han

**Affiliations:** 1 Nephrology, Kidney Disease Center, The First Affiliated Hospital, College of Medicine, Zhejiang University, Hangzhou, CHN

**Keywords:** retrospective analysis, adverse events, remission rate, iga nephropathy, hydroxychloroquine

## Abstract

Background:Hydroxychloroquine (HCQ) influences both toll-like receptor (TLR) signaling and leukocyte activation, which are speculated to play a role in the pathogenesis of IgA nephropathy (IgAN).

Methods:This is a single-centered retrospective study involving 426 IgAN patients diagnosed from May 2016 to August 2020. All patients were matched according to a propensity score matching (PSM) to produce three groups: renin-angiotensin-aldosterone system inhibitors (RAASi) group (RAASi only), corticosteroids group (corticosteroids only or combined with RAASi), and HCQ group (HCQ only or combined with RAASi), consisting of 63 patients for each group.

Results: After PSM, the median urine protein/creatinine ratio (UPCR) of overall patients was 0.91 g/g, while their median serum creatinine was 87.00 μmol/L. After the median follow-up period of 11.03 months, the total remission rates of the RAASi group, corticosteroids group, and HCQ groups were 49.21% (n = 31), 74.60% (n = 47), and 52.38% (n = 33), respectively (p = 0.017). Thirteen (6.88%) patients experienced a decline in estimated glomerular filtration rate (eGFR) of more than 25% from baseline, including six (9.52%) patients in the RAASi group, three (4.76%) patients in the corticosteroids group, and four (6.35%) patients in HCQ group (p = 0.677). One (1.59%) patient in the HCQ group had blurred vision and continued to use HCQ after ruling out retinal lesions by ophthalmic examination.

Conclusion: HCQ is effective in inducing remission and well-tolerated in IgAN patients with mild to moderate proteinuria.

## Introduction

Immunoglobulin (Ig) A nephropathy (IgAN) is a prevalent glomerular disease characterized by the deposition of IgA1-containing immune complexes in the mesangial area. Galactose-deficient IgA1 (Gd-IgA1) has been identified as a potentially pathogenic abnormality in IgAN [1,2]. A portion of IgAN patients progress to end-stage renal disease (ESRD), and proteinuria serves as an important prognostic factor. Hydroxychloroquine (HCQ) has anti-inflammatory and immunomodulating properties and has been widely used in autoimmune diseases. Recent studies have demonstrated that HCQ effectively alleviated persistent proteinuria in IgAN patients [3-5]. Liu et al. conducted a randomized, placebo-controlled study in 60 IgAN patients under optimized renin-angiotensin-aldosterone system inhibitors (RAASi) therapy, revealing that HCQ significantly reduced proteinuria at six months compared to placebo [4]. Gao et al. conducted a retrospective analysis of 28 IgAN patients and found that HCQ therapy ameliorated proteinuria within 24 weeks, although it was not statistically different than losartan [6]. Yang et al. further compared the efficacy of HCQ in 92 IgAN patients with 92 historical controls undergoing corticosteroid therapy and reported that the percent of decline in proteinuria at six months was not significantly different between the HCQ group and corticosteroids group [7].

In this study, we retrospectively analyzed IgAN patients with mild to moderate urine protein, dividing them into three groups according to their treatments, namely RAASi alone, corticosteroids, or HCQ. We aimed to evaluate the efficacy and safety of HCQ in comparison to RAASi or corticosteroids and build a predictive model in remission in IgAN patients.

## Materials and methods

We reviewed the data of patients diagnosed with IgAN from May 2016 (the time when we began using HCQ for these patients) to August 2020 in the Kidney Disease Center of the First Affiliated Hospital of Zhejiang University School of Medicine. The inclusion criteria were IgAN patients who (1) had regular follow-up for more than six months with no change in treatment during this period, (2) were treated with no other immunosuppressive agents except corticosteroids and HCQ or no combination use of corticosteroids and HCQ, (3) had a baseline urine protein/creatinine ratio (UPCR) ≥ 0.5 g/g and baseline estimated glomerular filtration rate (eGFR) ≥ 45 ml/min/1.73 m^2^. The exclusion criteria were IgAN patients who (1) did not receive any of the three drugs, namely RAASi, corticosteroids, and HCQ; (2) had severe infection, active hepatitis, tumor, pregnancy, or other renal diseases. The patients were followed up until February 2021.

All patients received optimized doses of RAASi as baseline treatment, except those with hypotension or other contraindications. According to treatment, patients were divided into the RAASi group (RAASi only), the corticosteroids group (corticosteroids only or combined with RAASi), and the HCQ group (HCQ only or combined with RAASi).

Using the electronic medical records, we collected clinical and laboratory data at admission (baseline data were collected before biopsy and treatment) and during follow-up, especially one, two, three, and six months after treatment and at the last visit. The eGFR was calculated using the chronic kidney disease epidemiology collaboration (CKD-EPI) equation. Laboratory results, remission rates, and adverse events were recorded until February 2021. We compared changes in UPCR, hematuria, serum creatinine (SCr), eGFR, serum albumin, serum uric acid (SUA), triglycerides (TG), and total cholesterol (TC) among the groups while calculating remission rates at six months and at the end of follow-up.

Histopathological lesions grading was based on the newly revised Oxford classification method in 2017, known as MEST-C [8]. It incorporates mesangial hypercellularity (M), endocapillary cellularity (E), segmental sclerosis (S), interstitial fibrosis/tubular atrophy (T), and crescents (C) into the MEST-C score.

Complete remission (CR) was defined as the disappearance of clinical symptoms and signs with UPCR less than 0.2 g/g accompanied by stable renal function (eGFR decrease < 25%). Partial remission (PR) was defined as the improvement or disappearance of clinical symptoms and signs accompanied by stable renal function, UPCR decreased by >50% and to a level of <1 g/g [9].

Quantitative data that conformed to a normal distribution were expressed as the mean ± standard deviation (SD), and non-normally distributed data were expressed as median (Q25, Q75). Categorical data were summarized as counts and percentages. To reduce baseline characteristics bias among the three groups, propensity score matching (PSM) was performed. Matching was carried out 1:1 without replacement, and a caliper width with a 0.25 SD was specified. Student’s t-test was employed for those with normal distribution and homogeneous variance, while non-parametric test was used for those with non-conformity. The chi-square test and Fisher's exact test were used for classification variables in the form of constituent ratio or rate (%). Differences at each stage were compared, and data that conformed to a normal distribution were compared using repeated measures analysis of variance, while data that did not follow a normal distribution were compared using non-parametric tests such as the Friedman test. Kaplan-Meier survival curves and Mann-Whitney U test were used to examine drug treatment patterns, and log-rank tests were used to compare the survival rate among the three groups. Factors associated with remission were identified after multivariate stepwise backward Cox regression analysis, including all relevant clinical variables. The variance inflation factors (VIFs) were used to check the collinearity among variables of interest. A nomogram was constructed to predict the non-remission possibility of IgAN patients based on statistically significant factors identified by the multivariate stepwise backward Cox regression model. The predictive performance of the nomogram was evaluated using the concordance index (C-index), calibration curve, and the areas under the receiver operating characteristic (ROC) curves.

All probabilities were two-tailed, and the level of significance was set at 0.05. Statistical analyses were performed with IBM SPSS Statistics version 27.0 (IBM Corp., Armonk, NY) and R statistical software version 4.3.1 (https://www.r-project.org/).

## Results

**Figure 1:**
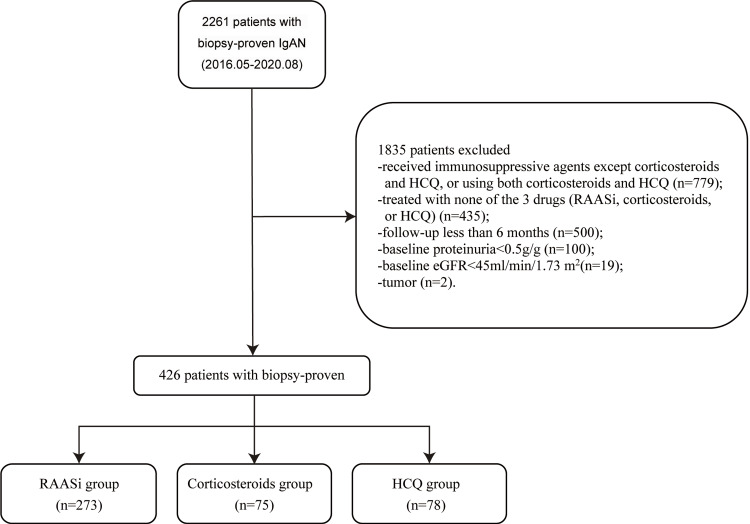
The flow chart of patient screening and enrollment IgAN: IgA nephropathy; HCQ: Hydroxychloroquine; RAASi: Renin-angiotensin-aldosterone system inhibitors; eGFR: Estimated glomerular filtration rate.

We screened a total of 2261 patients diagnosed with IgAN through clinical presentations and renal biopsy in our center. The flow chart detailing the study design is presented in Figure 1.

After inclusion, 426 patients (Table 1) were included with 273 patients in the RAASi group, 75 patients in the corticosteroids group, and 78 patients in the HCQ group. Their median age was 35.00 (28.00, 45.00) years. Males accounted for 44.13% (n = 188) of the patient population. Their baseline UPCR was 0.89 (0.65, 1.28) g/g, and eGFR was 85.93 (67.51, 108.34) ml/min/1.73 m^2^. Most patients (88.03%, n = 375) were treated with RAASi.

**Table 1:** Baseline characteristics of 426 IgAN patients The data has been represented as n (%), median (Q25, Q75), etc. IgAN: IgA nephropathy; RAASi: Renin-angiotensin-aldosterone system inhibitors; UPCR: Urine protein/creatinine ratio; TG: Triglycerides; TC: Total cholesterol; eGFR: Estimated glomerular filtration rate; SCr: Serum creatinine; M: Mesangial hypercellularity; E: Endocapillary cellularity; S: Segmental sclerosis; T: Interstitial fibrosis/tubular atrophy; C: Crescents; n: Number.

	IgAN (n = 426)
Male, n (%)	188 (44.13)
Age, years	35.00 (28.00, 45.00)
Disease duration, months	12.00 (2.00, 24.00)
Follow-up, days	411.00 (257.00, 719.25)
RAASi therapy, n (%)	375 (88.03)
Baseline urinary blood cell count, /μL	78.75 (28.98, 256.88)
Baseline UPCR, g/g	0.89 (0.65, 1.28)
Baseline serum albumin, g/L	42.80 (39.50, 45.70)
Baseline TG, mmol/L	1.44 (0.98, 2.17)
Baseline TC, mmol/L	4.48 (3.92, 5.11)
Baseline eGFR, ml/min/1.73 m^2^	85.93 (67.51, 108.34)
Baseline SCr, μmol/L	85.00 (64.00, 104.00)
MEST-C score of renal pathology	
M (0/1)	382/44
E (0/1)	408/18
S (0/1)	93/333
T (0/1/2)	396/28/2
C (0/1/2)	297/129/0

Comparative characteristics of these three groups are detailed in Table 2. There were significant differences among the groups on male proportion, age, disease duration, follow-up time, RAASi therapy (but no difference between the corticosteroids group and HCQ group), and baseline characteristics including urinary blood cell count, serum albumin, eGFR, and TG. There was no significant difference in the scores of mesangial hypercellularity (M), endocapillary cellularity (E), segmental sclerosis (S), and interstitial fibrosis/tubular atrophy (T) among the three groups. The ratio of crescent formation (C) was lower in the RAASi group than in the other two groups. At six months, the CR rate of the RAASi group, corticosteroids group, and HCQ group was 19.29% (n = 49), 38.03% (n = 27), and 22.22% (n = 16), respectively, while the total remission rates were 45.67% (n = 116), 63.38% (n = 45), and 52.78% (n = 38), respectively (p = 0.005). At the end of follow-up, the CR rates of the RAASi group, corticosteroids group, and HCQ group were 29.30% (n = 80), 40.00% (n = 30), and 26.92% (n = 21), respectively, while the total remission rates were 54.58% (n = 149), 72.00% (n = 54), and 57.69% (n = 45), respectively (p = 0.031). Further pairwise comparisons showed that the CR and total remission rates of the corticosteroids group were significantly higher than those of the RAASi group at six months and at the end of follow-up.

**Table 2:** Clinical characteristics and outcomes of the RAASi group, corticosteroids group, and HCQ group The data has been represented as n (%), median (Q25, Q75), etc. RAASi: Renin-angiotensin-aldosterone system inhibitors; HCQ: Hydroxychloroquine; UPCR: Urine protein/creatinine ratio; TG: Triglycerides; TC: Total cholesterol; eGFR: Estimated glomerular filtration rate; SCr: Serum creatinine; M: Mesangial hypercellularity; E: Endocapillary cellularity; S: Segmental sclerosis; T: Interstitial fibrosis/tubular atrophy; C: Crescents; PR: Partial remission; CR: Complete remission; n: Number. ^a^Compared with the RAASi group, p < 0.05. ^b^Compared with the corticosteroids group, p < 0.05.

	RAASi group (n = 273)	Corticosteroids group (n = 75)	HCQ group (n = 78)	P-value
Male, n (%)	133 (48.72)	31 (41.33)	24 (30.77)^a^	0.016
Age, years	37.00 (31.00, 47.00)	31.00 (25.00, 40.00)^a^	32.00 (27.00, 41.00)^a^	<0.001
Disease duration, months	12.00 (3.00, 36.00)	6.00 (0.80, 24.00)^a^	18.00 (4.00, 36.00)	0.021
Follow-up, days	553.00 (315.00, 930.00)	306.00 (231.00, 448.00)^a^	289.00 (221.75, 433.50)^a^	<0.001
RAASi therapy, n (%)	273 (100)	44 (58.67)^a^	58 (74.36)^a^	<0.001
Baseline urinary blood cell count, /μL	68.50 (24.60, 224.50)	121.20 (41.68, 337.48)	101.80 (38.15, 384.80)	0.038
Baseline UPCR, g/g	0.89 (0.64, 1.28)	0.91 (0.65, 1.51)	0.90 (0.68, 1.18)	0.746
Baseline serum albumin, g/L	42.80 (39.50, 45.70)	41.10 (36.23, 45.18)	43.60 (41.05, 46.50)^b^	0.006
Baseline TG, mmol/L	1.40 (0.95, 2.25)	1.66 (1.24, 2.30)	1.22 (0.93, 1.78)^b^	0.010
Baseline TC, mmol/L	4.45 (3.91, 5.13)	4.64 (4.01, 5.63)	4.48 (3.87, 4.93)	0.309
Baseline eGFR, ml/min/1.73 m^2^	85.16 (70.78, 104.91)	78.13 (61.19, 106.46)	92.60 (66.99, 116.82)^b^	0.036
Baseline SCr, μmol/L	83.00 (67.00, 103.00)	92.00 (72.50, 109.25)	79.00 (59.75, 99.25)	0.053
MEST-C score of renal pathology				
M (0/1)	247/26	68/7	67/11	0.479
E (0/1)	263/10	72/3	73/5	0.563
S (0/1)	69/204	13/62	11/67	0.063
T (0/1/2)	257/15/1	67/7/1	72/6/0	0.339
C (0/1/2)	213/60/0	42/33/0^a^	42/36/0^a^	<0.001
At six months of follow-up				0.005
PR	67/254 (26.38%)	18/71 (25.35%)^a^	22/72 (30.56%)	
CR	49/254 (19.29%)	27/71 (38.03%)^a^	16/72 (22.22%)	
Total remission	116/254 (45.67%)	45/71 (63.38%)^a^	38/72 (52.78%)	
At the end of follow-up				0.031
PR	69/273 (25.28%)	24/75 (32.00%)^a^	24/78 (30.77%)	
CR	80/273 (29.30%)	30/75 (40.00%)^a^	21/78 (26.92%)	
Total remission	149/273 (54.58%)	54/75 (72.00%)^a^	45/78 (57.69%)	

IgAN patients in the three groups were matched for characteristics including sex, age, RAASi therapy, urinary blood cell count, serum albumin, TG, and eGFR by 1:1 PSM. After PSM, there were 63 patients in each group, with no statistically significant difference among the three groups, except for the follow-up time and RAASi therapy (but no difference between the corticosteroids group and the HCQ group). The characteristics of the three groups after matching are shown in Table 3. The patients in the corticosteroids group had a median prednisone dose of 20.00 (20.00, 28.75) mg/d at the beginning. The patients in the HCQ group had a median HCQ dose of 6.35 (5.08, 7.14) mg/kg at the beginning.

**Table 3:** Clinical characteristics and outcomes of the RAASi, corticosteroids, and HCQ groups after PSM The data has been represented as n (%), median (Q25, Q75), etc. PSM: Propensity score matching; RAASi: Renin-angiotensin-aldosterone system inhibitors; HCQ: Hydroxychloroquine; UPCR: Urine protein/creatinine ratio; TG: Triglycerides; TC: Total cholesterol; eGFR: Estimated glomerular filtration rate; SCr: Serum creatinine; M: Mesangial hypercellularity; E: Endocapillary cellularity; S: Segmental sclerosis; T: Interstitial fibrosis/tubular atrophy; C: Crescents; PR: Partial remission; CR: Complete remission; n: Number, VIF: Variance inflation factor. ^a^Compared with the RAASi group, p < 0.05.

	RAASi group (n = 63)	Corticosteroids group (n = 63)	HCQ group (n = 63)	P-value
Male, n (%)	22 (34.92)	26 (41.27)	20 (31.75)	0.526
Age, years	37.00 (29.00, 46.00)	31.00 (25.00, 40.00)	32.00 (28.00, 41.00)	0.092
Disease duration, months	6.00 (2.00, 15.00)	10.00 (0.80, 24.00)	18.00 (4.00, 36.00)	0.261
Follow-up, days	581.00 (315.00, 953.00)	294.00 (230.00, 399.00)	288.00 (214.00, 421.00)^a^	<0.001
RAASi therapy, n (%)	63 (100.00)	40 (63.49)^a^	45 (71.43)^a^	<0.001
Baseline urinary blood cell count, /μL	76.70 (28.90, 284.20)	116.75 (40.53, 326.03)	107.00 (41.40, 419.10)	0.596
Baseline UPCR, g/g	0.94 (0.68, 1.40)	0.88 (0.64, 1.29)	0.90 (0.66, 1.22)	0.788
Baseline serum albumin, g/L	42.30 (37.68, 44.73)	42.65 (39.43, 45.38)	42.90 (40.55, 46.10)	0.211
Baseline TG, mmol/L	1.36 (0.97, 2.27)	1.60 (1.11, 2.03)	1.22 (0.93, 1.95)	0.096
Baseline TC, mmol/L	4.70 (4.14, 5.21)	4.51 (3.94, 5.11)	4.42 (3.84, 4.89)	0.194
Baseline eGFR, ml/min/1.73 m^2^	83.60 (66.78, 103.44)	78.13 (61.19, 105.76)	92.26 (64.72, 113.87)	0.215
Baseline SCr, μmol/L	81.50 (67.50, 102.00)	92.50 (72.50, 110.25)	81.00 (60.50, 100.50)	0.155
MEST-C score of renal pathology VIF				
M (0/1)	53/10	59/4	57/6	0.209
E (0/1)	59/4	61/2	59/4	0.776
S (0/1)	16/47	8/55	7/56	0.060
T (0/1/2)	60/3/0	58/4/1	58/5/0	0.714
C (0/1/2)	47/16/0	37/26/0	36/27/0	0.079
At six months of follow-up				0.034
PR	15/51 (29.41%)	19/57 (33.33%)	17/53 (32.08%)	
CR	9/51 (17.65%)	19/57 (33.33%)	9/53 (16.98%)	
Total remission	24/51 (47.06%)	38/57 (66.66%)	26/53 (49.06%)	
At the end of follow-up				0.017
PR	16/63 (25.40%)	25/63 (39.68%)^a^	18/63 (28.57%)	
CR	15/63 (23.81%)	22/63 (34.92%)^a^	15/63 (23.81%)	
Total remission	31/63 (49.21%)	47/63 (74.60%)^a^	33/63 (52.38%)	

**Figure 2:**
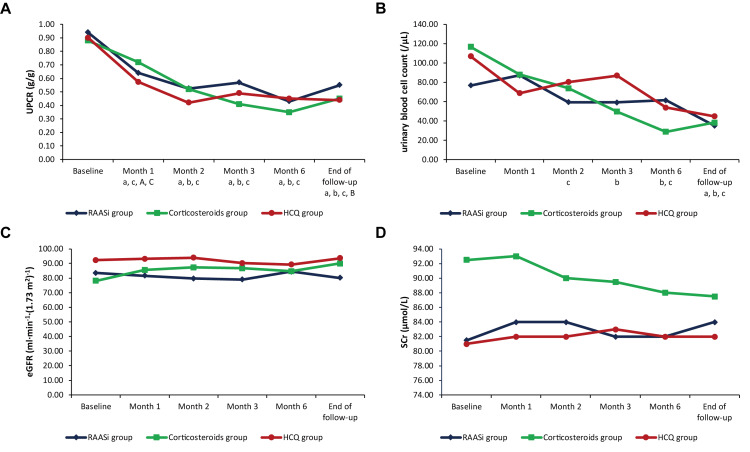
Changes in UPCR (A), urinary blood cell count (B), eGFR (C), SCr (D) during follow-up in the RAASi, corticosteroids, and HCQ groups after PSM RAASi: Renin-angiotensin-aldosterone system inhibitors; HCQ: Hydroxychloroquine; UPCR: Urine protein/creatinine ratio; eGFR: Estimated glomerular filtration rate; SCr: Serum creatinine; PSM: Propensity score matching. ^a, b, c^: p < 0.05 compared with baseline in the RAASi, corticosteroids, and HCQ groups, respectively. ^A, B, C^: p < 0.05 of each time-point compared with the one preceding it in the RAASi, corticosteroids, and HCQ groups, respectively.

**Figure 3:**
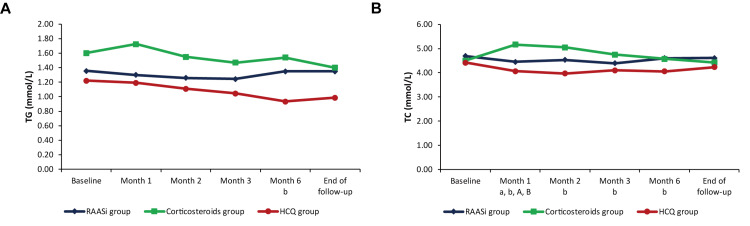
Changes in TG (A) and TC (B) during follow-up in the RAASi, corticosteroids, and HCQ groups after PSM RAASi: Renin-angiotensin-aldosterone system inhibitors; HCQ: Hydroxychloroquine; TG: Triglycerides; TC: Total cholesterol; PSM: Propensity score matching. ^a, b^: p < 0.05 compared with baseline in the corticosteroids and HCQ groups, respectively. ^A, B^: p < 0.05 of each time-point compared with the one preceding it in the corticosteroids and HCQ groups, respectively.

During follow-up, UPCR and urinary blood cell count tended to decrease and were significantly different from baseline in all three groups. The eGFR and SCr levels kept generally stable in all three groups (Figure 2). However, in the corticosteroids group, UPCR rebounded at the end of follow-up (0.45 [0.19, 0.85] g/g) compared with that at six months (0.35 [0.15, 0.52] g/g; p = 0.033) (Figure 2, Panel A). In terms of lipid profiles, patients in the corticosteroids group had significantly higher TC in the first few months of follow-up than baseline, while the patients in the HCQ group had a trend of decrease in TC levels during follow-up (Figure 3, Panel B).

**Figure 4:**
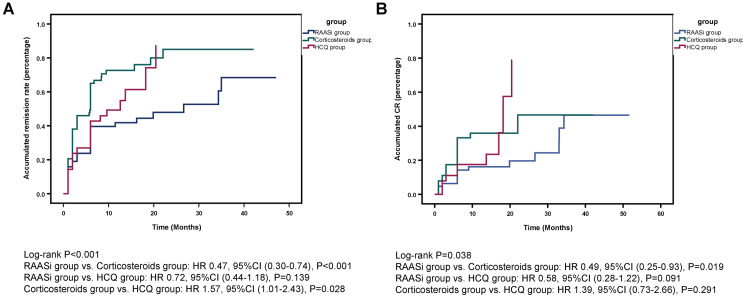
Accumulated total remission (A) and CR (B) rates in the RAASi, corticosteroids, and HCQ groups after PSM RAASi: Renin-angiotensin-aldosterone system inhibitors; HCQ: Hydroxychloroquine; HR: Hazard ratio; CI: Confidence interval.

The remission rates of the three groups after PSM are shown in Table 3. At six months, the CR rates of the RAASi group, corticosteroids group, and HCQ group were 17.65% (n = 9), 33.33% (n = 19), and 16.98% (n = 9) respectively, while the total remission rates were 47.06% (n = 24), 66.66% (n = 38), and 49.06% (n = 26), respectively (p = 0.034). At the end of the follow-up, the CR rates of the RAASi group, corticosteroids group, and HCQ group were 23.81% (n = 15), 34.92% (n = 22), and 23.81% (n = 15), respectively, while the total remission rates were 49.21% (n = 31), 74.60% (n = 47), and 52.38% (n = 33), respectively (p = 0.017). The remission of the corticosteroids group was significantly better than that of the RAASi group. Kaplan-Meier analysis showed that the total remission (p < 0.001) and CR (p = 0.038) rates were statistically different among the three groups (Figure 4). Further pairwise comparisons showed that the CR rate in the corticosteroids group was significantly higher than the RAASi group (p = 0.019) (Figure 4, Panel B).

Next, we performed multivariate Cox regression analyses to determine independent predictors of remission. We put the factors including male proportion, age, disease duration, RAASi therapy, baseline characteristics including urinary blood cell count, UPCR, serum albumin, TG, TC, eGFR, SCr, and MEST-C score of renal pathology into stepwise backward Cox regression analysis. The results showed that treatment, age, and urinary blood cell count were independent predictors for remission (Table 4). There was no collinearity between these variables (VIFs from 1.01 to 1.16).

**Table 4:** Multivariate analysis for remission of IgAN patients after PSM HR: Hazard ratio; CI: Confidence interval; RAASi: Renin-angiotensin-aldosterone system inhibitors; HCQ: Hydroxychloroquine; PSM: Propensity score matching.

Variable	Multivariable p-value	HR	95% CI
Treatment			
RAASi	1 (Reference)		
Corticosteroids	<0.001	2.746	1.696-4.445
HCQ	0.120	1.505	0.899-2.519
Age	0.034	1.020	1.002-1.039
Urinary blood cell count	0.013	1.000	1.000-1.000

**Figure 5:**
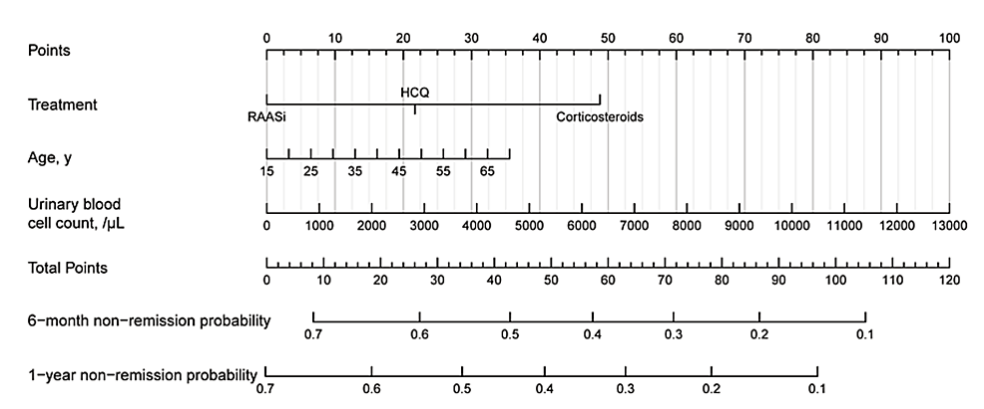
A nomogram for predicting remission of IgA nephropathy after treatment RAASi: Renin-angiotensin-aldosterone system inhibitors; HCQ: Hydroxychloroquine.

Based on the multivariate regression analysis, a nomogram model for IgAN was constructed (Figure 5). The value of each variable was calculated using the nomogram. The total points projected on the bottom scales indicate the non-remission probabilities of six months and one year.

**Figure 6:**
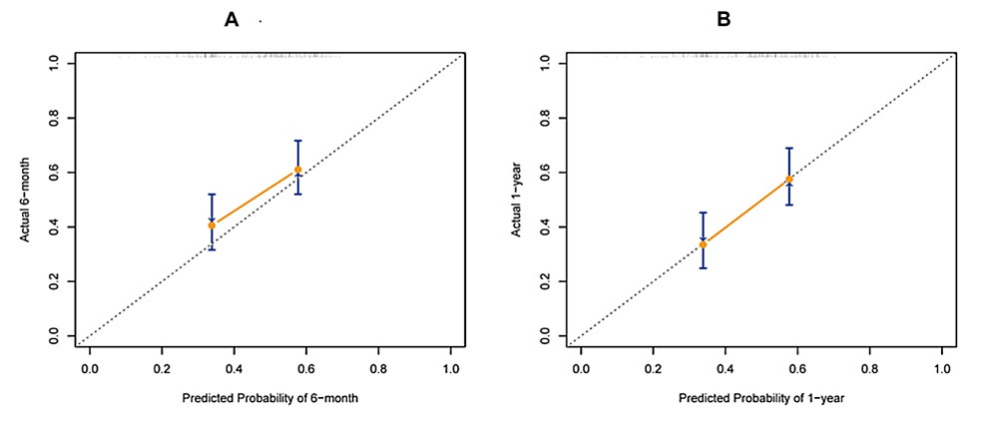
The calibration curves for predicting patient non-remission probability at six months (A) and one year (B) in the internal verification

**Figure 7:**
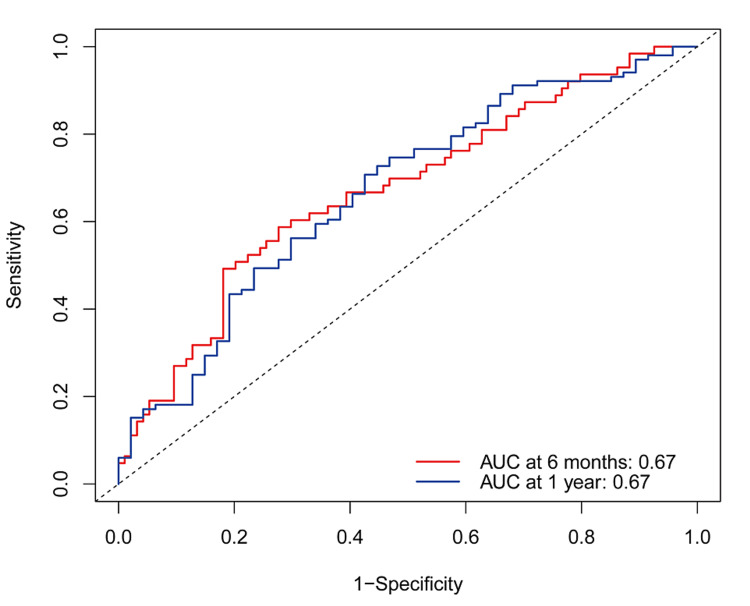
Performance of nomogram to predict non-remission probability in IgAN AUC: Area under curve; IgAN: IgA nephropathy.

Internal verification showed that the concordance index (C-index) value was 0.615 (95% CI: 0.555-0.676). The calibration curve demonstrated good concordance between the predicted and observed values for non-remission probability at both six months and one year (Figure 6). Additionally, the ROC analysis revealed that the nomogram’s area under the curve (AUC) value was 0.67 at six months and one year (Figure 7).

The adverse events in the three groups after PSM are summarized in Table 5. Thirteen (6.88%) patients experienced a decline in eGFR of more than 25% from baseline, including six (9.52%) in the RAASi group, three (4.76%) in the corticosteroids group, and four (6.35%) in the HCQ group (p = 0.677). Additionally, one (1.59%) patient in the corticosteroids group had abnormal liver function, and one (1.59%) experienced gastrointestinal hemorrhage. Furthermore, one (1.59%) patient in the HCQ group had blurred vision and continued to use HCQ after undergoing an ophthalmic examination that excluded retinal lesions.

**Table 5:** Adverse events in the RAASi, corticosteroids, and HCQ groups after PSM RAASi: Renin-angiotensin-aldosterone system inhibitors; HCQ: Hydroxychloroquine; n: Number; PSM: Propensity score matching.

	RAASi group (n = 63)	Corticosteroids group (n = 63)	HCQ group (n = 63)	P-value
Worse of renal function, n (%)	6 (9.52)	3 (4.76)	4 (6.35)	0.677
Gastrointestinal hemorrhage, n (%)	0 (0.00)	1 (1.59)	0 (0.00)	1.000
Abnormal liver function, n (%)	0 (0.00)	1 (1.59)	0 (0.00)	1.000
Blurred vision, n (%)	0 (0.00)	0 (0.00)	1 (1.59)	1.000

## Discussion

There have been several reports on the treatment of IgAN using HCQ [4-7]. Two studies compared the efficacy of HCQ and RAASi in IgAN patients [5,6]. In a prospective cohort, 28 IgAN patients were treated with three months of losartan and then matched into two groups, the HCQ group (treated with HCQ plus losartan) and the control group (treated with losartan alone). After 24 weeks, HCQ significantly reduced proteinuria and achieved a higher remission rate than the control group [6]. Another retrospective cohort showed that HCQ plus RAASi achieved a greater proportion of patients with more than 30% reduction in proteinuria at six months than RAASi alone [5]. A randomized double-blind controlled trial showed that 50% of patients in the HCQ group had more than 50% reduction in 24-hour urinary protein at six months [4]. These remission rates were similar to the results in our study and demonstrated that HCQ could effectively reduce proteinuria in IgAN patients.

Studies of lipid profiles suggest that TC increased significantly from baseline after one month under corticosteroid therapy but decreased significantly in the HCQ group. The findings match the lipid-lowering effect of HCQ observed in earlier studies. Many clinical studies have found that HCQ can significantly reduce TG [3,10] and TC [10-13]. Qiao et al. indicated that HCQ reduced adipogenesis through the peroxisome proliferator-activated receptor gamma (PPARγ) pathway [14].

This study found that HCQ was well-tolerated, but it is important to know that it can have side effects that cannot be ignored. Previous studies have confirmed that HCQ can cause retinal damage [15], although the incidence rate is low and the damage is irreversible once it occurs. In this study, one patient in the HCQ group experienced blurred vision, but no retinal damage was detected, which may be due to the short follow-up period and relatively low dose used. Studies have shown that most reported cases of poisoning occur in patients who have been using HCQ for more than seven years or in those whose cumulative dose exceeds 1000 grams of HCQ [16]. Literature analysis of initial HCQ prescription dosing between 2007 and 2016 found that the dose of HCQ had dropped sharply according to the weight-based guidelines of ophthalmology [17]. For patients with chronic kidney disease, current guidelines do not recommend reducing the dosage of HCQ [18,19].

As a statistical prediction model, a nomogram can visually display the relevant indicators that affect the remission in multivariate stepwise Cox regression analysis and predict the remission rate by a simple calculation. The results showed that hematuria was significantly associated with remission in IgAN patients. Hematuria is a common symptom in IgAN, but the relationship between hematuria and prognosis is still controversial. Previous studies have found that the presence of hematuria could indicate active renal inflammation [20]. Sevillano et al. showed that revealed time-averaged hematuria was one of the independent predictors of ESRD, while resolution of hematuria improves IgAN outcomes [21]. This finding is consistent with our results relating to hematuria. Previous studies have also used nomograms to predict the prognosis of IgAN patients with excellent predictive performance, but most of them were used to predict recurrence [22], crescent formation [23], renal function decline [24], or the occurrence of ESRD [24,25].

However, the remission rate of the HCQ group was not as high as that of the corticosteroids group at six months, and there was no significant difference compared to RAASi. The low remission rate may be due to the short follow-up time. Previous literature pointed out that the efficacy of HCQ was related to the course of treatment [26]. The recommended dose of HCQ by Kidney Disease: Improving Global Outcomes (KDIGO) guidelines is 6.5 mg/kg/d or 400 mg/d.

There are limitations to be acknowledged in this study. This study is a retrospective and single-center study. Although we may provide data in clinical practice, the effects of these treatments were not well compared. The median follow-up time in our cohort was 11.03 months, and it may not show long-term efficacy and relapse. So, in the current stage, we still cannot recommend the use of HCQ in IgAN. It is necessary to have more proof based on prospective randomized controlled trials (RCTs) or real-world studies. In addition, the C-index of the nomogram established in this study was relatively low, indicating that the nomogram needs more corrections based on large, long-term observation data. Further external validation is needed.

## Conclusions

Based on the pharmacological action, HCQ has shown potential as a treatment for IgAN. The study found that HCQ is effective in inducing remission and well-tolerated in IgAN patients with mild to moderate proteinuria, exhibiting similar efficacy to RAASi but being inferior to corticosteroids. Notably, HCQ had only a minimal impact on lipid levels in IgA patients, unlike corticosteroids which are known to potentially cause adverse changes in lipid profiles. This could be a beneficial aspect of HCQ treatment, especially for patients with pre-existing lipid abnormalities. To further establish the long-term effectiveness and potential for relapse, future studies should include a larger population and longer follow-up periods. This will provide a more comprehensive understanding of HCQ's efficacy and its potential role in the management of IgAN.
